# Use of Contrast-Enhanced Ultrasound in Carotid Atherosclerotic Disease: Limits and Perspectives

**DOI:** 10.1155/2015/293163

**Published:** 2015-06-21

**Authors:** Gianfranco Varetto, Lorenzo Gibello, Claudio Castagno, Simone Quaglino, Matteo Ripepi, Emilio Benintende, Andrea Gattuso, Paolo Garneri, Stefano Zan, Giacomo Capaldi, Ugo Bertoldo, Pietro Rispoli

**Affiliations:** Division of Vascular Surgery, Department of Surgical Sciences, University of Turin, Azienda Ospedaliera Universitaria Città della Salute e della Scienza, Molinette Corso Bramante 88, 10126 Torino, Italy

## Abstract

Contrast-enhanced ultrasound (CEUS) has recently become one of the most versatile and powerful diagnostic tools in vascular surgery. One of the most interesting fields of application of this technique is the study of the carotid atherosclerotic plaque vascularization and its correlation with neurological symptoms (transient ischemic attack, minor stroke, and major stroke) and with the characteristics of the “vulnerable plaque” (surface ulceration, hypoechoic plaques, intraplaque hemorrhage, thinner fibrous cap, and carotid plaque neovascularization at histopathological analysis of the sample after surgical removal). The purpose of this review is to collect all the original studies available in literature (24 studies with 1356 patients enrolled) and to discuss the state of the art, limits, and future perspectives of CEUS analysis. The results of this work confirm the reliability of this imaging study for the detection of plaques with high risk of embolization; however, a shared, user-friendly protocol of imaging analysis is not available yet. The definition of this operative protocol becomes mandatory in order to compare results from different centers and to validate a cerebrovascular risk stratification of the carotid atherosclerotic lesions evaluated with CEUS.

## 1. Introduction

CEUS represents one of the major breakthroughs in the field of diagnostic ultrasound. In fact, the contrast medium, injected intravenously, passes through the vascular region of interest generating an enhanced ultrasound signal that allows a better morphological and functional imaging resolution [1]. Moreover the simplicity and rapidity of execution even at the patient's bedside encouraged its application for different vascular purposes: the definition of the degree of stenosis and plaque surface (i.e., higher sensitivity for plaque ulceration or near-occlusion stenosis), the diagnosis of intrastent restenosis, the detection of type II endoleaks (even those with low flow rate), the assessment of organ perfusion (i.e., kidney transplantation), or the assessment of tumor perfusion (important prognostic parameter in metastatic tumors) [2]. Another interesting field of application of CEUS is the study of the carotid atherosclerotic plaque vascularization and its correlation with cerebrovascular neurological events (stroke, transient ischemic attack). Data available from large study population in literature reveal a 5-year risk for ipsilateral stroke of 5% of patients with asymptomatic carotid artery stenosis of 70% or greater [3–5]. The traditional parameters for the description of a carotid atherosclerotic plaque (degree of stenosis, systolic peak velocity) are insufficient predictors of the risk of embolization while the vascularization of the atherosclerotic plaque, evaluated with CEUS, is correlated with a more accurate “qualitative” analysis of the carotid disease [2]. Differently from what happens for the other fields of application of CEUS, the study of the carotid plaque vascularization requires a more precise quantification of the enhancement which is obtained by a visual or semiautomated method [6]. The objective of this review is to analyze the application of CEUS for the study of plaque microvascularization in carotid atherosclerosis and to define the strengths and limits of this technique.

## 2. Correlation between Plaque Microvascularization and Potentially Symptomatic Atherosclerotic Lesions

**Figure 1:**
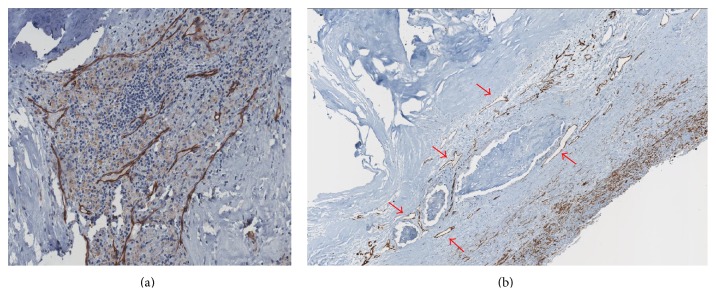
(a) Histopathological preparation of a carotid atherosclerotic plaque colored with CD31 endothelial-specific stain (brown) showing the close correlation between plaque vascularization and the inflammatory response. (b) Histopathological preparation of a carotid atherosclerotic plaque colored with CD31 endothelial-specific stain (brown) showing larger microvessels (red arrows) and areas of plaque hemorrhage.

The inflammatory etiopathogenesis of atherosclerosis has been widely demonstrated in several animal models and later confirmed in human models [7]. In particular at the level of flow turbulence along the vascular tree (vessel bifurcation), the subintimal deposition of cholesterol and oxidized lipoproteins generates an inflammatory response with the recruitment of white blood cells, primarily macrophages, and the production of cytokines and enzymes. Among different cellular responses to the inflammatory stimulus there is the liberation from the smooth muscles cells of vascular endothelial growth factor (VEGF) with the consequent activation of neoangiogenesis of the vessel wall (Figure 1). The newly formed vessels inside the atherosclerotic plaque however are immature and leaky due to reduced gap junctions, thus serving as a port of entry for other inflammatory cells, lipids, and even red blood cells, which contribute to plaque growth. In the same contest macrophages produce metalloproteinases, like MMP-9 and other collagenases that destroy the connective fibrous tissue, thus stimulating the neovessels growth [8]. All these plaque changes lead to a vulnerable atherosclerotic plaque [7]. It is common experience in clinical practice that irregular, ulcerated plaque surfaces, lipid necrotic core, thin fibrous cap, anechoic-hypoechoic appearance, and intraplaque neovessels characterize potentially unstable atherosclerotic lesions with high risk of embolization and thrombosis. However, it is common experience that not all the atherosclerotic lesions behave in the same way. In fact some of them react to the inflammation stimulus with the precipitation of calcium salts (calcific plaques) and others with the simple transformation of muscular cells into connective cells (fibrous plaques). The exact mechanism of differentiation of the plaque is still partially unknown but some factors, such as genetic predisposition, uncontrolled risk factors like smoke, diabetes mellitus, hypertension, and dyslipidemia, may influence the process.

## 3. Methods

### 3.1. Search Strategy

This review included all available original studies reporting the use of CEUS for the evaluation of the vascularization of the carotid atherosclerotic plaque and its correlation with ipsilateral neurological events and with other indicators of plaque “vulnerability”. Data were collected from the online MEDLINE database in July 2014 using PubMed (National Center for Biotechnology Information, US National Library of Medicine, Bethesda, MD). The search strategy included the words “carotid,” “atherosclerosis,” and “contrast-enhanced.” No time restriction for publication date was used. The search was restricted to articles published in English and to studies in humans.

### 3.2. Study Selection, Data Extraction, and Analysis

All abstracts were reviewed online and articles meeting the inclusion criteria were identified and downloaded for data extraction. In addition, a manual search of the reference lists of the identified studies was performed, and references were evaluated. Data collected from the selected studies were registered into a specific database and analyzed with Microsoft Excel 2010 (Microsoft Corporation, Redmond, WA). Despite the heterogeneity of the studies it was possible to create different macrocategories of correlation between the degree of the enhancement of the atherosclerotic lesion and neurological symptoms (transient ischemic attack, minor stroke, and major stroke) or other plaque characteristics: histology (quantification of the microvascularization of the carotid plaque after surgical removal), echogenicity with Doppler ultrasound (evaluated with a visual assessment according to the Gray Weale scale—GW scale—or with a software analysis according to the Gray Scale Median—GSM), signs of microembolization, or plaque instability (plaque surface ulceration, cerebral ipsilateral microembolization detected with transcranial Doppler ultrasonography in absence of other possible causes).

## 4. Results

The search identified 24 original studies (100% single-center studies) suitable for revision from 2007 to 2014 (Table 1) [9–32]. The review population consisted of 1356 patients with carotid atherosclerosis examined with CEUS; 946 patients (70%) were asymptomatic. In 19 studies (76%) the contrast medium used was SonoVue (Bracco, Altana Pharma, Konstanz, Germany), 2 studies (7%) used Definity (Bristol-Myers Squibb Medical Imaging, Billerica, Massachusetts), and 3 studies (7%) used Optison (GE Healthcare, Little Chalfont, Buckinghamshire, UK). CEUS imaging of the carotid artery was performed using linear array vascular probe with transmission frequencies ranging from 3 to 15 MHz and mechanical index ranging from 0.06 to 1.4. The analysis of the contrast enhancement was performed with a semiautomated software in 16 studies (often home-made software). In 15 studies, a visual classification of the plaque enhancement was performed by two different operators (nonuniformity in scoring methods). In 7 studies (29%) plaques were evaluated with both methods. The correlation between data obtained with CEUS and histopathologic results was performed in 12 studies (50%, 433 patients) and all the studies found a statistical significant correlation: plaques with higher enhancement have a highly significant vascularization of the plaque. Ten studies (41%, 578 patients) evaluated the correlation between CEUS images and the presence of ipsilateral neurological symptoms: in three cases (30%, 91 patients) results did not reach statistical significance to demonstrate that plaques with greater contrast enhancement are more frequently related to clinical symptoms. In 7 studies (29%, 331 patients) CEUS analysis was compared to the plaque echogenicity; all the studies found a statistically significant correlation: plaques with high contrast enhancement have a low echogenicity. Six studies (25%, 351 patients) compared CEUS with other indicators of “vulnerable plaque” (surface ulceration, cerebral microembolization detected with transcranial Doppler); all the studies found a statistically significant correlation: the increased vascularization of carotid atherosclerotic plaques evaluated with CEUS is more frequently related to cerebral microembolization or surface ulceration. One study (4%, 143 patients) compared CEUS to the patient's gender with a statistically significant correlation (*P* = 0.03): women have a higher contrast enhancement of the carotid plaque.

## 5. Discussion

### 5.1. Correlation between CEUS and the Characteristics of the Vulnerable Carotid Plaque

Data collected from an overall review population of 1356 patients demonstrate that information obtained by CEUS imaging is strictly dependent not only on the plaque microvascularization (histological analysis) but also correlated to the plaque echogenicity, the surface ulceration, and the intraplaque hemorrhage (100% agreement among the studies). All these parameters together define an atherosclerotic plaque with high risk of embolization. In detail plaques with low echogenicity, surface ulceration, and histopathological findings of intraplaque hemorrhage have a greater enhancement with CEUS compared to calcific or fibrous plaques. Results on the correlation between CEUS and clinical neurological symptoms do not reach statistical evidence in all the studies; in fact 7 studies (487 patients) established a significant relationship between the two parameters while 3 studies (91 patients) did not reach statistical significance. The reason of this finding is not completely clear: according to the general agreement among the studies on the reliability of CEUS for the detection of a “vulnerable plaque,” it would be logical to expect also a correlation with neurological symptoms. However these results could be partially explained with the low single-center study population or with silent neurological damage among asymptomatic patients. From the results obtained, CEUS appears to be one of the most reliable imaging studies for the detection of atherosclerotic lesions with high risk of embolization because of the correlation with every single expression of the plaque instability (intraplaque hemorrhage, surface ulceration, low echogenicity, and plaque microvascularization). In current literature there is not a specific indication for the “suitable patient” for CEUS analysis; however, the strong relationship between the plaque enhancement and the echogenicity of the carotid plaque led some authors to select a subgroup of the population (asymptomatic with an hypoechoic carotid plaque) that would benefit most from this investigation [19].

### 5.2. CEUS Imaging Analysis

The acquisition of CEUS images was made with different ultrasound hardware and different presettings (i.e., mechanical index, linear probes). The majority of the studies used a linear probe with frequencies between 3 MHz and 10 MHz. The mechanical index as well differs among the studies; however, many authors agree that a lower mechanical index (between 0.06 and 0.2) is preferred to obtain a better image resolution and to reduce the risk of rupture of the microbubbles of contrast agent [33].

The analysis of the images obtained with CEUS can be performed in two ways: the semiautomated method and the visual score of the enhancement; often they are used together. The semiautomated assessment is performed with a software (usually a home-made software) that analyzes the variation of enhancement intensity over time in a region of interest (ROI). The timing of the analysis differs among the studies (i.e., evaluation of maximum signal intensity [19], evaluation of the late phase of contrast enhancement [23]) as well as the unit of measurements (i.e., dB-enhanced [20], percentage ratio between area of plaque captation and silent areas [19]). Strengths of this method are a better reproducibility over time with the same software and presetting and the reduction of the operator-related bias. Some limits of this method are the need of reprocessing the images (not immediate result) and the need of a motion tracking algorithm (not simple implementation) to reduce errors of interpretation on the luminal side of the plaque. On the other hand, the visual score allows a direct interpretation of the CEUS examination (easier applicability in a clinical setting) but with an increased risk of operator-related bias and less accurate confrontation of the results obtained. Moreover a univocal visual scale is still not defined. Currently no data are available on the superiority of the semiautomated analysis compared to the visual assessment for CEUS. From the data collected in literature it becomes mandatory to create a generally shared operative protocol for the interpretation of CEUS results in order to compare experiences from different centers.

### 5.3. Tolerability of CEUS

The contrast medium injected intravenously consists of microbubbles filled with gasses (air or high molecular weight gasses) and it has been shown to have a good safety profile. In literature the three most common side effects observed in clinical trials were headache (2,3%), injection site pain (1,4%), and injection site bruising, burning, or paresthesia (1,7%) [34]. Among the studies evaluated in this review, 19 used SonoVue (Bracco), 3 used Optison (GE Healthcare), and 2 studies performed CEUS with Definity (Bristol-Myers). None of the studies reported severe side effects, procedural complication, or anaphylactic reactions to the contrast medium.

### 5.4. Limits and Future Perspectives of CEUS for the Study of Carotid Atherosclerosis

Despite the excellent results described above, some limits are still evident. Firstly CEUS remains an operator dependent imaging technique; for this reason the creation of a common semiautomated software for the image elaboration could increase the reproducibility and the homogeneity among different operators. Moreover the sensibility of CEUS decreases in heavy calcified plaques with important acoustic shadow. Lastly the bidimensional analysis of the plaque enhancement assumes that the longitudinal cross section of the plaque analyzed is representative of the whole carotid plaque. For this reason it could be interesting to apply the emerging technique of 3D and 4D Doppler ultrasonography to CEUS for a global evaluation of the carotid atherosclerotic lesion.

## 6. Conclusion

This review confirms the great potential of CEUS for the detection of carotid atherosclerotic plaques with high risk of embolization. However the different procedures used for the analysis of the contrast enhancement limit the possibility to compare results from different centers. The creation of a common, well-established, user-friendly, and operative protocol is essential to overcome this limit to create multicentric studies in order to define a cerebrovascular risk stratification with accurate enhancement cut-offs.

## Figures and Tables

**Table 1:** Summary of studies on carotid contrast-enhanced ultrasound (CEUS) for the detection of intraplaque neovascularization. The results of each study are expressed with the statistical significance (*P* value) of the correlation between the parameters.

Author	Year	Number of patients	Symptomatic	Asymptomatic	Histology	Contrast agent	Ultrasound apparatus	Vascular probe	Mechanical index	CEUS analysis		Results					
										Semiautomated analysis	Visual assessment	CEUS and histology	CEUS and carotid symptoms	CEUS and echogenicity	CEUS and other vulnerable characteristics	CEUS and gender	Software and visual assessment
Li et al. [9]	2014	17	13	4	1	SonoVue	Philips iU-22 ultrasound system	L 9-3	0,07	1	1	0,002	0	0	0	0	0

Hjelmgren et al. [10]	2014	13	7	6	0	SonoVue	Siemens S2000 system	L 9-4	0,06	1	1	0	0,05	0,01	0	0	0,001

Kim et al. [11]	2014	89	0	89	1	SonoVue	Acuson Sequoia 512 Siemens system	L 15-8	0,19	0	1	0,021	0	0	0	0	0

van den Oord et al. [12]	2014	143	0	143	0	SonoVue	Philips iU-22 ultrasound system	L 9-3	0,06–0,08	1	1	0	0	0	0	0,03	0

Müller et al. [13]	2014	33	17	16	1	SonoVue	Antares Siemens system	nn	nn	1	1	0,01	Not significant	0	0	0	0,02

Vavuranakis et al. [14]	2013	14	0	14	1	SonoVue	Acuson Sequoia 512 Siemens system	L 15-8	nn	1	0	0,002	0	0	0	0	0

Ritter et al. [15]	2013	41	41	0	0	SonoVue	GE Logiq 7 system	L 9-3	nn	0	1	0	0	0	0,02	0	0

Hjelmgren et al. [16]	2013	52	10	42	0	SonoVue	Siemens S2000 system	L 9-4	0,06	1	0	0	Not significant	0,02	0	0	0

van den Oord et al. [17]	2013	69	0	69	0	SonoVue	Philips iU-22 ultrasound system	L 9-3	0,06–0,08	1	1	0	0	0	0,05	0	0

Zhou et al. [18]	2013	46	24	22	0	SonoVue	Acuson Sequoia 512 Siemens system	L 2	0,07	0	1	0	Not significant	0	0,05	0	0

Varetto et al. [19]	2012	51	12	39	1	SonoVue	Esaote MyLab 25 Gold system	L 9-2	nn	1	0	0,001	0,01	0,02	0	0	0

Faggioli et al. [20]	2011	22	7	15	1	SonoVue	Philips iU-22 ultrasound system	L 9-3	0.13	1	0	0,003	0,006	0	0	0	0

Hoogi et al. [21]	2011	27	8	19	1	Definity	Philips iU-22 ultrasound system	L 8-4		1	0	0,01	0	0	0	0	0

Shalhoub et al. [22]	2011	31	16	15	1	SonoVue	Philips iU-22 ultrasound system	L 12-5	0,34	1	0	0,004	0	0	0	0	0

Owen et al. [23]	2010	37	16	21	0	SonoVue	Philips iU-22 ultrasound system	L 12-5	0,34	1	0	0	0,005	0	0	0	0

Huang et al. [24]	2010	183	86	97	0	SonoVue	Acuson Sequoia 512 Siemens system	L 15-8	0,35	1	1	0	0,001	0	0	0	0,001

Staub et al. [25]	2010	147	17	130	0	Definity	ATL HDI 5000 Philips system	L 7-4	0,06–0,1	0	1	0	0	0	0,034	0	0

Giannoni et al. [26]	2009	77	64	9	1	SonoVue	Acuson Sequoia 512 Siemens system	L 9-3	0,4–1,4	1	1	>0,05	0,001	0,001	0	0	0

Magnoni et al. [27]	2009	25	0	25	0	Optison	GE Vivid 7 system	L 7	0,08–01	0	1	0	0	0	0,001	0	0

Xiong et al. [28]	2009	104	35	69	0	SonoVue	GE Logiq 9 system	L 9-3	0,13	1	0	0	0,001	0	0	0	0

Huang et al. [29]	2008	63	0	63	0	SonoVue	Acuson Sequoia 512 Siemens system	L 15-8	0,35	1	0	0	0	0,01	0	0	0

Coli et al. [30]	2008	32	28	4	1	Optison	GE Vivid 7 system	L 7	0,08–0,1	0	1	0,005		0,001	0	0	0

Vicenzini et al. [31]	2007	23	0	23	1	SonoVue	Acuson Sequoia 512 Siemens system	L 6-15	0,4–1,4	0	1	>0,05	0	>0,05	>0,05	0	0

Shah et al. [32]	2007	17	5	12	1	Optison	ATL HDI 5000 Philips system	L 7-4	0,06–0,1	0	1	0,002	0	0	0	0	0

Total		1356	406	946	12					16	15						
